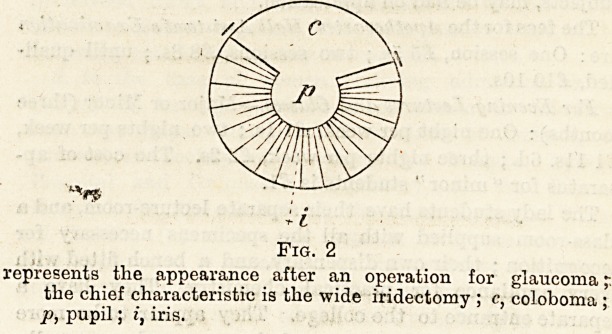# The Hospital. Nursing Section

**Published:** 1903-09-05

**Authors:** 


### The Hospital.

**Nursing Section.**

Contributions for this Section of "The Hospital" should be addressed to the Editor, "The Hospital" Nursing Section, 28 & 29 Southampton Street, Strand, London, W.C.

No. 884.—Vol. XXXIV. *SATURDAY, SEPTEMBER* 5, 1903.

### Notes on News from the Nursing World.

### OUR CHRISTMAS DISTRIBUTION.

With regard to our Christmas distribution of useful articles of clothing for the patients in hospitals and infirmaries, which we desire this year to be able to extend to several institutions which have not hitherto received parcels, it may be worth while to state that the garments most acceptable are warm petticoats and underclothing for the women, shirts and knitted socks for the men, and flannel garments, frocks, stockings and socks for the children. Of course there are other things, but those mentioned are always so welcome that the supply, however liberal, cannot be equal to the demand. There is no doubt that patients like to think that the work is done by nurses themselves, and this sentiment is sure to be respected by our kind helpers. But there is no reason why every nurse who addresses herself to the task of making garments should not induce her friends to render assistance. Some who may not really have time to [do the work themselves can purchase the materials, or get others to purchase them. The primary object is, of course, to swell the number of articles sent in, and we only throw out these suggestions thus early lest there should be among our readers nurses who fancy that unless they can both provide the materials and make the articles most needed with their own hands, in their own often hard-earned leisure, their contributions are not thoroughly appreciated.

### A MATERNITY HOSPITAL FOR HONG KONG.

An extremely interesting ceremony occurred in Hong Kong on July 26th, when Lady Blake, wife of the Governor, laid the foundation stone of the new Alice Memorial Maternity Hospital. This is an extension of the Alice Memorial and Nethersole Hospitals, which were erected some years ago. The credit of originating it is due to Mrs. Stevens, who for twelve years has been matron of the existing hospitals. Unhappily, she was too ill to attend the function which but for her might not have taken place; and one of the speakers, in paying a tribute to the value of her efforts and her devotion to her work, stated that there was no hope of her recovery. As the result of her practical experience, Mrs. Stevens came to the conclusion that a separate maternity hospital was essential, and, largely in consequence of the generous support given by the Chinese residents, the greater portion of the necessary money has already been obtained. It is anticipated that the building will be finished by December 31st. It will be in charge of Dr. Sibree, a lady with the Edinburgh diploma, who goes out to Hong Kong under the auspices of the London Missionary Society, the governing body of the hospital; and the nursing staff will consist of the matron and two nurses. Lady Blake, in performing the opening ceremony, said that throughout China there is great need for maternity hospitals.

### SELF-SACRIFICE OF A BIRMINGHAM NURSE.

A striking illustration of modest heroism was recently given by a nurse at the Jaffray Branch Hospital, Erdington. The story is told by a former patient, who says that in the same ward as himself was a little boy who had been dreadfully burnt about the legs and body. He had been in the Jaffray Hospital about six months, besides a period in the General Hospital, and his skin showed no signs of healing, so the sister of the ward allowed the doctor to take 24 pieces of skin from her arm for grafting on the boy's burns. During the operation the brave nurse, he states, did not show any signs of discomfort, but was all smiles and was joking with the other patients and cheering up the little boy. She also assisted the doctor by holding up the skin of her arm whilst he cut it away. The fact that this was not the first time the nurse had allowed the same thing to be done, shows that her action was not the result of a sudden impulse, but was prompted by a fixed principle.

### THE NURSING STAFF AT NEWTON ABBOT.

The nursing question came before the Newton Board of Guardians again at their last meeting. Attention was called to the fact that two of the bedrooms in the new nurses' home were not sufficiently ventilated, and the matter was referred to the Building Committee. There were 18 applications for the two vacancies on the nursing staff. A proposal that three of the candidates ahould be asked to appear before the board was rejected in favour of an amendment that two should be appointed forthwith. In view of the frequent changes at Newton Abbot Infirmary, we think that it would have been more satisfactory if the Guardians had decided to see the nurses before engaging them.

### THE AGE QUESTION AT DEVONPORT.

The Devonport Board of Guardians came to a decision last Friday which brings them into direct conflict with the Local Government Board. It seems that the Local Government Board had declined to assent to the appointment of a probationer nurse because she was under 21 years of age. The infirmary committee of the Devonport board, however, resolved that the young lady, "being over 18 years of age," having shown an aptitude for the work, and having performed her duties so far to the satisfaction of the Board of Guardians, the medical officer, and the superintendent nurse, it was desirable to confirm her appointment, and "that the Local Government Board be informed that in future the Guardians would bear in mind their suggestion as to age." This resolution the Guardians at their meeting adopted, and are therefore apparently bent upon defying the authorities at Whitehall. We can understand their attitude, but we cannot understand that of the superintendent nurse, who, as a practical woman, must be perfectly well aware that the age-limit for probationers in general hospitals or workhouse infirmaries could not safely be lowered to 18. She might not have been able to prevent the Guardians from arriving at an unwise conclusion, but if she had expressed herself opposed upon principle to the employment of a probationer of 18, she would at any rate have been free from all responsibility for the retrograde policy which the Devonport Guardians wish to pursue.

### VICTORIAN TRAINED NURSES' ASSOCIATION.

The half-yearly examination conducted by the Conjoint Board of Examiners was held at the Melbourne Hospital, and at the sub-centres, Ballarat District Hospital and Bendigo Gold District General Hospital, in June, the practical examination following. Thirty candidates presented themselves, and 21 passed. The examination of nurses before the Conjoint Board will not be compulsory until July, 1905, except for hospitals containing fewer than 40 beds. But so far, since the formation of the Victorian Trained Nurses' Association, only two institutions have preferred to conduct their own final examinations.

### NURSING OPHTHALMIA IN SCHOOLS.

It will not be possible to form any judgment as to the wisdom of the course which the Metropolitan Asylums Board have decided to take in reference to the children under their charge who require to be treated for ophthalmia, until the building works at the White Oak School and the High Wood School have been completed and in working order for some little time. But the method of control which it is proposed to carry out seems judicious. The Board have adopted a scheme under which each cottage will be governed by a house-mother, untrained, who will be responsible to the matron only, and not to the charge nurse, for the children while "at home." The idea is to keep the spheres of duty of the house-mother and the charge nurse quite distinct, and it is quite correctly assumed that no trained charge nurse would care to undertake the multifarious duties which would naturally fall upon the cottage-mothers.

### MATERNITY TRAINING FOR SEA-GOING NURSES.

There is one point of importance in connection with the movement for securing the appointment of nurses on ocean-going ships which those who wish to take up such work would do well to bear in mind. It is essential that nurses who wish to join should have received maternity training. We are hopeful that some of the leading steamship companies will have the wisdom to decide at an early date that the presence of a trained nurse is necessary to the proper equipment of their vessels.

### FOR DELICATE LADIES OF LIMITED MEANS.

There is a Home at Torquay which deserves to be well known, because it provides for the wants of delicate ladies of limited means. We refer to Erith House Institution, which was founded many years ago under the management of a ladies' committee, and has attached to it a lady superintendent and a trained nurse, as well as a staff of servants. It is visited by several of the principal medical men of the town, and the payments are on a very moderate scale. The Home, which is in the best part of Torquay, and is surrounded by large grounds, opens in October and closes in June.

### LOCHRANZA NURSING ASSOCIATION.

An interesting feature in connection with the Lochranza Nursing Association, was a concert held in the school house and given by the inhabitants of and visitors to Lochranza. The chair was taken by the Rev. Robert Kerr, who made an excellent and capable president. Pianoforte solos, songs, and recitations were given, with success by each performer. The proceeds amounted to £8 10s., quite a record collection. This result will encourage the subscribers in their efforts to support a Queen's nurse for the district.

### NURSES' QUARTERS AT SOUTH SHIELDS.

The South Shields Guardians have had under consideration two proposals for making proper provision for the probationers in course of training at the workhouse infirmary. They have rejected the plan for accommodating the nurses in the infirmary building, and have adopted the scheme for erecting a nurses' home outside. This decision was arrived at almost entirely owing to the difference in cost, the estimate for the necessary extensions to the infirmary being about £4,000, and that for the separate quarters amounting to £3,580.

### MERTHYR TYDFIL WORKHOUSE INFIRMARY.

Having further considered the application of the Merthyr Tydfil Board of Guardians, who asked that the workhouse infirmary might be recognised as a major training school for nurses, the Local Government Board have intimated that they cannot depart from the rule which they have laid down, viz., that the medical officer of the workhouse must be resident. It remains for the Merthyr Guardians to determine whether, in order to secure for their infirmary the position they desire, it is not possible to fulfil the only condition which is lacking.

### SHORT ITEMS.

Miss E. S. Mason, Miss M. M. Rees, and Miss M. Walker, have been appointed staff nurses of Queen Alexandra's Imperial Military Nursing Service. — In future St. Mary's Nursing Home (Fulham Midwifery School), Parsons Green, will be under the charge of Miss Bessie M. Worrall, as matron. Miss Worrall was for over seven years matron of the Manchester Maternity Hospital, two years sister midwife at Queen Charlotte's Lying-in Hospital, and two years at the Birkenhead Lying-in Hospital.

### The Nursing Outlook.

"From magnanimity, all fear above;
From nobler recompense, above applause,
Which owes to man's short outlook all its charm."

### NURSING EXHIBITIONS.

There are few nurses at private work who do not rejoice at the chance of seeing an exhibition of nursing appliances and hygienic novelties; but whereas in England we have constant small shows of this sort, in connection with the Congress of the Sanitary Institute and so on, in America the scheme is carried to a perfection that probably only those who visited the World's Fair at Chicago can realise. There is going to be an Exposition at St. Louis next year which promises to beat all previous records, and we heartily wish that at least a few English nurses may find time to compete, and also to go and see. The nursing comes under the Department of Social Economy, and there are five classes which appeal to the nurse. Class 784, Destitute, Neglected, and Delinquent Children; 785, Institutional Care of Destitute Adults; 786, Care and Relief of Needy Families in their Homes; 787, Hospitals, Dispensaries, and Nursing; 788, the Insane, Feeble-minded, and Epileptic.

With regard to the children, the nurses in Poor-law schools, in elementary schools, in ringworm and ophthalmic schools might have much to say. Under this heading there is to be a "gallery of eminent philanthropists and reformers," and there surely should the picture of Mary Carpenter have a place. Then the Workhouse Nursing Association should stand under the class of Care for Destitute Adults, and the Queen Victoria Jubilee Nursing Institute under the class including "Home" Care of the Needy. The photographs of nurses in boats, on bicycles, on horses and donkeys, going to their work at Achil, Labrador, St. Kilda, or other outlying spots would be a very telling exhibit. Then here are some of the sub-headings for exhibits in Class 787: Relief on the Battle-field; Sterilising; Diet Kitchen; Hospitals for Consumptives; Maternity Hospitals; Leper Colonies; Dispensaries; The Evolution of the Nurse; Gallery of Famous Nurses; The Visiting Nurse; Nurse's Handwork; Training Schools for Nurses. And in the next class: Evolution of the Hospital for the Insane; County Asylums; After-care Societies; Training Schools for Attendants; The Effects of Epilepsy on the Mind; Epileptic Colonies; Evolution of the Treatment of the Feeble-minded; Institution Management; Gallery of Eminent Workers.

But perhaps the most hopeful note of all in the pamphlet from the Advisory Committee of the Exposition is that "Suggestions of any character will be cheerfully appreciated by this department in its attempt to make this the greatest and most effective exhibit of its kind ever presented to the public." It only remains to remark that the office is in Victoria Street, Westminster, that the committee for England has been appointed, and that there is no nurse on that committee. In other countries the nursing authorities are already preparing, but apparently no move has been made in England. To those of us who can remember the time when the United States pleaded for Miss Alice Fisher to go out and organise trained nursing at Blochley, who can remember when the doors of St. Thomas's were besieged by folk from far lands begging for Nightingale nurses to go abroad and carry the knowledge of trained nursing to many countries, there is a little soreness in recognising that the children are beating the mother, that the skill in which England was at one time pre-eminent is now developing more rapidly in the Colonies, on the Continent, and in the new worlds; and that in questions of hygiene and diet, we could even learn much from Japan. And when Italy and Japan wanted help to organise trained nursing it was to America they turned and not to England.

Besides the St. Louis Exposition next year there is the International Congress of Nurses at Berlin; the first will appeal to the eye, the second to the ear, but there will be much to be learnt from both. That is to say, much could be learnt from both if three or four picked nurses were sent to each, and subsequent arrangements were made for the large body of English nurses to profit by the experience of the travellers. If a nursing exhibit were sent out to St. Louis under the charge of two nurses, these women would have an opportunity not only of seeing what other countries sent, but also of meeting the nurses of many lands, and the doctors of all countries, who will be visiting the exhibition. To travel for many days and at great expense to see a nursing exhibition, or join in a conference, or attend at the meeting of a society, is quite the natural thing in the eyes of an American enthusiast. To have models made of all that strikes her as worth copying, to collect photographic albums of institutions, appliances, even uniforms, to gather specimens and found a scientific library of the nursing books in many languages—these are part of her professional work. And therefore it is that there is a bigger and more charitable view of life and greater professional keenness and skill about those nurses who have taken part in previous exhibitions and conferences. It is necessary, while watching with care to produce a sufficient supply of hard-working, well-trained individual nurses to meet the needs of the public, not to forget also to develop the intellectual and organising nurse when found; for it is in her hands that will rest the advance of the profession, not only in the eyes of England, but in the eyes of the whole world.

### Lectures on Ophthalmic Nursing.

By A. S. COBBLEDICK, M.D., B.S.Lond., Senior Clinical Assistant and late House-Surgeon and Registrar to the Royal Eye Hospital.

### LECTURE XVIII. — DIAGNOSIS, PROGNOSIS AND TREATMENT OF ACUTE GLAUCOMA.—CHRONIC GLAUCOMA.

THE diagnosis was touched upon in Lecture XVII., when speaking of acute iritis, but the importance of the subject is so great that nothing will be lost through repetition.

Acute glaucoma is most frequently confused with acute iritis; the chief points to be borne in mind in making a diagnosis are the condition of the intra-ocular tension, the pupil, the iris and anterior chamber. In acute glaucoma the tension is very much increased, the pupil is dilated and oval in shape, the iris is not much altered in colour, and the anterior chamber is almost obliterated; whereas in acute iritis the tension is for the most part unaltered or diminished, the pupil is small and circular, the iris altered in colour and the free edge roughened with exudation, and the anterior chamber if anything is deeper than normal.

The following table sums up these points:—

| — | ACUTE GLAUCOMA. | ACUTE IRITIS. |
|---|---|---|
| Tension .. .. | Much increased | Normal or diminished |
| Pupil .. .. | Dilated and oval | Small and circular |
| Anterior Chamber | Practically abolished | Normal or deepened |
| Iris .. .. .. | Colour unaltered | Colour deepened |

Occasionally a case of acute iritis is met with with very considerable increase of intra-ocular tension; when this occurs in a dark iris, and a mydriatic has been used without the surgeon's knowledge, there may be a real difficulty in coming to a correct conclusion. Other conditions where the white sclerotic is more or less red, as in acute conjunctivitis, scleritis, etc., the tension of the eye, the pupil and iris are normal, and with ordinary care no confusion should occur.

*Prognosis.*—This largely depends on the time that has elapsed before the case is seen by the surgeon. If seen early, and submitted to immediate operation, the trouble is cut short and valuable sight is retained; on the other hand, if the case has been neglected, the prognosis in regard to good vision is bad, although the eye can be saved.

Much also depends on the severity of the attack; a malignant attack may destroy all vision but perception of light in a few hours; slighter attacks, with a moderate increase of intra-ocular tension, retain fair central vision for some days; in fact, some of these slighter cases, if totally untreated, settle down in a week, but usually develop a more acute attack during the next few months.

*Treatment.*—This consists in immediate operation in all cases: if vision is to be saved it can only be effected by early operation, not to be postponed longer than would an operation for strangulated hernia. The operation is iridectomy—that is, removal of a segment of the iris, or sclerotomy.

If one is not fully equipped for the radical operation, and it is most important to lower the tension at once, what is termed a *scleral puncture* may be performed.

All that is required for this operation is a Græfe cataract knife and a pair of fixation forceps; it is an advantage to have an eye speculum but it is not indispensable. The point of the knife is passed through the sclerotic about a third of an inch behind the corneo-sclerotic margin in the region of the insertion either of the internal or external rectus tendon. When about a quarter of an inch of the blade has been passed through the sclerotic rotate it so that it is at right angles to the first position, it is then withdrawn; the result is that a small crucial incision is made through which a head of vitreous exudes; this serves to lower the tension and relieve the patient's pain. Another advantage is that within a few hours' time there is some semblance of an anterior chamber, so that, as will be seen, the major operation is rendered less difficult.

*The Major Operation.*—This is one of the few operations in ophthalmic surgery for which a general anæsthetic is frequently necessary; the parts are in such a state of tension and inflammation that cocaine does not take much effect, and it requires an exceptionally self-controlled patient to keep quiet enough for the few minutes necessary.

Chloroform is the best general anæsthetic, and before commencing to operate, the patient should be *deeply* under the anæsthetic; if he is not, it is more than likely that as soon as the sensitive iris is touched, he reflexly tries to close the eye, with the result that the lens and vitreous may be expelled and the visual use of the eye quite lost.

The instruments necessary for this operation are an eye speculum, a Græfe cataract knife (the blade should be short and thin, but firm), fixation forceps, iris forceps and scissors. All these instruments have been depicted in a previous lecture.

The instruments should be boiled in distilled water and placed on a sterilised cloth, or in an antiseptic lotion, *e.g.*, carbolic acid 1 in 40. Whilst the patient is being anæsthetised, the conjunctival sac is well irrigated with boracic lotion. After the speculum is applied the surgeon obtains a firm hold of the conjunctiva with the fixation forceps.

FIG. 1.—Right Eye.

*a*, point of entry of knife; *b*, point of counter puncture (the dotted line shows the remainder of the incision); *E*, external; *I*, internal; *p*, pupil; *i*, iris.

The point of the knife should enter the sclerotic immediately behind the corneo-scleral junction at the point *a* (Fig. 1), and at such an angle as to pass into what remains of the anterior chamber.

FIG. 2

represents the appearance after an operation for glaucoma; the chief characteristic is the wide iridectomy; *c*, coloboma; *p*, pupil; *i*, iris.

The difficulty is to pass the knife from *a* to *b* without injuring the lens, and this can only be accomplished by proceeding very slowly and keeping the point of the knife as far from the free edge of the iris as possible; when the point

*b* is reached, pass the point of the knife through the sclerotic and *very slowly* with a to and fro saw-like movement complete the section. The slower the section is completed the less likely is there to be any troublesome hæmorrhage.

When the section is completed the aqueous escapes, and more than likely the great intra-ocular pressure will cause the iris to bulge out of the wound; pick the iris up near the outer part of the incision and snip it away from its attachment until the portion corresponding to the section has been removed.

The only dressing necessary is an iced boracic flap, which must be changed frequently.

*Sclerotomy* is an operation advised by some. It consists in leaving the iris intact, and not quite completing the section, so as to leave a bridge of scleral tissue at the uppermost point of the section.

### Nurses and Dispensing.

### LONDON SCHOOLS OF PHARMACY.

(*Continued from Page* 279.)

### THE WESTMINSTER COLLEGE OF CHEMISTRY AND PHARMACY, LIMITED.

This institution was founded by Mr. George S. V. Wills in 1874, in order to provide for pharmaceutical, medical, dental, and other students a sound education in the scientific principles on which chemistry and the practice of pharmacy are based. Students are prepared for the examinations of the Pharmaceutical Society and the Assistants' Examination of Apothecaries' Hall. The principal is Mr. G. S. V. Wills, F.C.S., Ph.C., who is assisted by qualified demonstrators.

The building, situated at the corner of Trinity Street and Swan Street, Borough, was originally a Baptist chapel; the chapel itself now forms the lecture hall, the students occupying the pews, which are all numbered, and the lecturer the platform, or "rostrum." The gallery forms the laboratory, where the students carry out the practical work of the syllabus. The botanical specimens, salts, etc., are arranged on the walls of the lecture hall and laboratory, and there is a good collection of autograph prescriptions. In the laboratory, which is lighted from the roof, each student has his separate bench, supplied with his own Bunsen burner, washer and sink, locker, and set of reagents. Separated from the laboratory is the balance-room, where students work under supervision of a demonstrator. There is a reference library. Mr. Wills reserves a separate small laboratory for his own analytical work. The college is lighted by electricity. All large apparatus is provided free of charge. The fees, which are inclusive and payable in advance, are as follows :—

*Day Lectures and Classes.*—For the Major Examination (including lectures, classes, and laboratory instruction): One session ($3\frac{1}{2}$ months), £6 6s.; until qualified, £10 10s. For the Minor Examination: One course, £8 8s.; two courses, £12 12s.; until qualified, £15 15s. Special fees for practical chemistry only, part time, or for any one or more subjects, may be had on application.

The fees for the *Apothecaries' Hall Assistant's Examination* are: One session, £5 5s.; two sessions, £8 8s.; until qualified, £10 10s.

*For Evening Lectures and Classes.*—Major or Minor (three months): One night per week, £1 1s.; two nights per week, £1 11s. 6d.; three nights per week, £2 2s. The cost of apparatus for "minor" students is £1.

The lady students have their separate lecture-room, and a class-room supplied with all the specimens necessary for recognition; their own dispensary, and a bench fitted with every appliance for practical chemistry. They have a separate entrance to the college. They appear to be more keen in their work than many of the men, and are usually successful in examination. Mr. Wills' opinion is that in delicacy of touch they are specially adapted for employment as dispensers, an occupation which requires a light touch.

Lectures are given by the Principal twice a day, and the lantern is a special feature of the institution; it is used for botanical and other slides. For the few weeks preceding each examination special drill classes are held in which many of the questions likely to be asked by the examiners are gone over daily. Judging by the testimonials of successful students these classes are of special value.

At the close of each term a college examination is held, and medals and certificates are awarded. The prescriptions set are frequently taken from past papers set in the minor examination.

The correspondence method of preparation appears to be used to great advantage; by its means a student may shorten the time of necessary attendance at the college, and consequently lessen the ultimate expense. The preliminary, minor and major examinations of the Pharmaceutical Society are thus prepared for by country students who are in business, or who cannot afford to enter for the full time at the college. Two lectures are usually sent out once a week, and, if desired, specimen salts for analysis accompany these. The whole course may be extended over several years or condensed into a few months. A large collection of autograph prescriptions is also sent out, by means of which the student may become familiar with what is to form a large part of his or her work in the future.

Mr. Wills has compiled nearly 20 handbooks on Materia Medica, Volumetric Analysis, etc. In the garden of his private house at Croydon are grown botanical specimens for use in the school, and in the summer excursions are made to the various botanic gardens in London or to the country. Students are provided with passes to the Royal Society's Gardens, Regent's Park.

A register of situations is kept, and there seems to be no difficulty in finding employment for a duly-qualified man or woman. It is evident that a strong personal interest in his pupils is taken by Mr. Wills, and that he follows them up with practical help and advice in after life.

(*To be continued.*)

### To Nurses.

**We invite contributions from any of our readers, and shall be glad to pay for "Notes on News from the Nursing World," or for articles describing nursing experiences, or dealing with any nursing question from an original point of view. The minimum payment for contributions is 5s., but we welcome interesting contributions of a column, or a page, in length. It may be added that notices of appointments, entertainments, presentations, and deaths are not paid for, but that we are always glad to receive them. All rejected manuscripts are returned in due course, and all payments for manuscripts used are made as early as possible after the beginning of each quarter.**

### The Central Midwives Board.

### THE IMPORTANT SECTIONS.

THE rules framed by the Central Midwives Board have now been considered by the Privy Council, together with the draft and minority reports, and a representation from the General Medical Council. The schedule as approved for a period of three years has now been published.

The important sections are *B*, *C*, and *D*.

*B*.—REGULATING THE ISSUE OF CERTIFICATES AND THE CONDITIONS OF ADMISSIONS TO THE ROLL OF MIDWIVES.

1. Candidates must satisfy the Central Midwives Board that they have reached a sufficient standard of general education and submit the following documents, duly filled in and signed: (*a*) a certificate of birth, showing that the candidate is not under 21 years of age; (*b*) certificates to the effect that the candidate has undergone the training set forth in *C*1 (1), (2) and (3); (*c*) a certificate of good moral character in the form prescribed by the board.

2. Candidates must pass an examination as hereinafter set forth.

3. A candidate who has complied with the above requirements and has successfully passed the examination shall receive a certificate in the form set out in the schedule, and her name shall be entered by the secretary on the roll of midwives.

4. The names of all women admitted to the Roll of Midwives under Section 6 (1) and (2) of the Midwives Act shall be printed in one single list and in alphabetical order.

*C*.—REGULATING THE COURSE OF TRAINING AND THE CONDUCT OF EXAMINATIONS AND THE REMUNERATION OF THE EXAMINERS.

1. No person shall be admitted to an examination unless she produces certificates that she has undergone the following course of training, viz.:—

(1) She must have, under supervision satisfactory to the Central Midwives Board, attended and watched the progress of not fewer than twenty labours, making abdominal and vaginal examinations during the course of labour and personally delivering the patient.

(2) She must have to the satisfaction of the person certifying nursed 20 lying-in women during the 10 days following labour.

The certificates as to (1) and (2) must be in the form prescribed by the Central Midwives Board, and must be filled up and signed either by a registered medical practitioner or by the chief midwife, or, in the absence of such an officer, by the matron of an institution recognised by the board, or in the case of a Poor-law institution by the matron, being a midwife certified under the Midwives Act, or a superintendent nurse certified in like manner and appointed under the Nursing in Workhouses Order, 1897, and attached to such an institution, or by a midwife certified under the Midwives Act and approved by the board for the purpose.

(3) She must have attended a sufficient course of instruction in the subjects named below. (See Section 4 *C*.) No period of less than three months shall be deemed sufficient for the purpose. The certificate must be in the form prescribed by the Central Midwives Board, and must be filled up and signed by a registered medical practitioner recognised by the Board as a teacher.

2. Candidates who intend to present themselves for examination must send notice to the secretary of the Central Midwives Board at least three weeks before the date fixed for the examination to commence, accompanied by the certificates mentioned in *B* 1 and *C* 1, and by the payment of the fee of one guinea, or, in the event of the candidate having presented herself on a former occasion and having failed to pass, the fee of 15s.

3. Any candidate who during the examination shows a want of acquaintance with the ordinary subjects of elementary education may be rejected on that ground alone.

4. The examination shall be partly oral and practical and partly written, and shall embrace the following subjects:—(*a*) The elementary anatomy of the female pelvis and generative organs. (*b*) Pregnancy and its principal complications, including abortion. (*c*) The symptoms, mechanism, course, and management of natural labour. (*d*) The signs that a labour is abnormal. (*e*) Hæmorrhage: its varieties and the treatment of each. (*f*) Antiseptics in midwifery and the way to prepare and use them. (*g*) The management of the puerperal patient, including the use of the clinical thermometer and of the catheter. (*h*) The management (including the feeding) of infants, and the signs of the important diseases which may develop during the first ten days. (*i*) The duties of the midwife as described in the regulations. (*j*) Obstetric emergencies, and how the midwife should deal with them until the arrival of a doctor. This will include some knowledge of the drugs commonly needed in such cases, and of the mode of their administration. (See *E* 16.) (*k*) Puerperal fever: its nature, causes, and symptoms. The elements of house sanitation. The disinfection of person, clothing, and appliances.

5. Due notice shall be given of the examinations to be held under the Act.

6. The remuneration of the examiners shall be such as may from time to time be recommended by the Central Midwives Board and approved by the Privy Council.

*D*.—REGULATING THE ADMISSION TO THE ROLL OF WOMEN ALREADY IN PRACTICE AS MIDWIVES AT THE PASSING OF THE ACT.

1. Applications for admission to the Roll of Midwives under Section 2 of the Midwives Act must be made on the prescribed forms, and must be forwarded to the Central Midwives Board, together with such one or more of the following certificates as may be required.

2. In the case of women claiming admission on the ground of having obtained a certificate in midwifery from the Royal College of Physicians of Ireland, the Obstetrical Society of London, the Coombe Lying-in Hospital and Guinness's Dispensary, or the Rotunda Hospital for the Relief of the Poor Lying-in Women of Dublin, (*a*) either the original certificate on which the application is based, or, in the event of the original certificate having been lost, a voucher from the accredited secretary or other agent of the certifying body to the effect that the certificate was granted to the applicant on such and such a date; and (*b*) a certificate signed by a justice of the peace, minister of religion, or registered medical practitioner, or the secretary of an institution (approved by the Central Midwives Board) of which the applicant is a

THE CENTRAL MIDWIVES BOARD—*Continued.*

member, or is or was an employee, stating that the applicant is the person to whom the aforementioned certificate in midwifery was granted. The secretary of the Board shall, by comparison of the handwriting, or by such inquiry as he may think necessary, satisfy himself as far as possible of the applicant's identity. The application must be accompanied by the fee of 10s.

3. In the case of women claiming admission on the ground of having obtained a certificate in midwifery from any institution or examining body other than those specified in Section 2 of the Midwives Act, the certificate on which the application is based, together with satisfactory evicence, in the form prescribed by the Central Midwives Board, to the effect that before the certificate was granted the applicant had received a proper course of instruction and training (including personal attendance, under competent supervision, upon at least 20 cases during and after labour) and had passed an examination in midwifery and the duties of a midwife, and that the institution or examining body by which the certificate was granted considers the applicant at the present time to be a proper person to be admitted to the Midwives' Roll. The application must be accompanied by a fee of 10s. The applicant may be required to furnish other documents or particulars to enable the board to decide whether the application can be granted.

4. In the case of women claiming admission on the ground of having been in *bona-fide* practice as midwives for twelve months previous to July 31st, 1902, a certificate to the effect that the applicant has to the personal knowledge of the person signing been in *bona-fide* practice as a midwife for at least twelve months prior to July 31st, 1902, and that she is trustworthy, sober, and of good moral character. This certificate must be in the form given in the schedule, must be signed by a justice of the peace, minister of religion, registered medical practioner, or other person acceptable to the Board, and must be accompanied by a fee of 10s.

5. The certificates to be issued by the Board under this section will be in the prescribed forms.

*Note.*—No application for admission to the Roll of Midwives under Section 2 of the Midwives Act, 1902, can be received after the 31st day of March, 1905.

The remaining sections deal with—

*E.*—Regulating, supervising, and restricting within due limits the practice of midwives, including:—
 The duties of the midwife to the patient.
 Duties to child.
 General.
 Notification.

*F.*—Deciding the conditions under which midwives may be suspended from practice.

*G.*—Defining the particulars required to be given in any notice under Section 10 of the Act.

### Private Nursing in America.

BY AN ENGLISH SISTER.

Before coming to America I was many times told by Americans and others that there was a great field for nurses in this country. After three years of experience in Boston, I feel that I would like my English sisters to know just how I found it.

There is scope for nurses, but they must have American training to be successful. In many of the hospitals a post-graduate course of three months is given; an English nurse could take this, and in that time she would learn American methods, and might also get into favour with some visiting physician who would give her cases afterwards.

### Chances of Work.

After training there are no nursing institutions or co-operations that a nurse can enter. She must take a room and join a club or directory. The latter is practically an intelligence office, and as there are 500 trained and 200 untrained or partially-trained nurses on its books, the chances of an English nurse getting work are small. The club is better. It is on the same principle, but is newer, and a limited number of nurses is accepted. There is quite a large waiting list, however, so it is usually months before a nurse is accepted. The club rooms are attractive, most of the popular magazines and papers are to be found there (amongst them The Hospital), and lectures and afternoon teas brighten the lives of the nurses who are fortunate enough to be members. There are also a few living rooms in the apartment which nurses may rent, and which are cheaper than those in comfortable boarding-houses.

### Life in the American Room.

Very often three or four nurses room together, thus curtailing the expense. Life in those quarters is anything but pleasant. I have met American nurses who have waited six months before getting a case, and during that time have had to stay indoors most of the time. When registered you are not supposed to leave the house for more than an hour at a time. This becomes very trying, and a nurse's health and spirits suffer in consequence.

### Fees and Food.

The private nursing fees are large compared with English ones, but living is so much higher and clothes so much more expensive (no outdoor uniform is worn), that at the end of the year a nurse in England will have more money than an American unless she has had a long case and had no opportunity to spend.

A great many of the nurses here are Irish and Nova Scotian, and are of a lower class than the majority of nurses in large hospitals in England. Young American ladies do not seem to enter the profession as a vocation. The lack of freedom in a nurse's life does not appeal to their spirit of liberty, consequently nurses do not have the same standing as with us.

### The Question of Social Standing.

Many of the people do not seem to understand that a nurse can also be a lady. From observation I should say that dressmakers, milliners, and those engaged in other trades have a better standing socially than a nurse. But I think that this applies also to men in the medical and other professions; they do not receive the same deference as is paid to them on the other side.

What I have written simply applies to private nursing. I have had a good hospital position offered to me (providing I took the post-graduate course) and also district work; but to those who think their work trying and their remuneration small, and who hanker after America and large fees, I would say—stay at home.

### Wants and Workers.

Will any nurse forward The Hospital the Monday after publication in return for postage pre-paid. Address Nurse Moore, Batheaston, Bath.

### Everybody's Opinion.

A NURSE'S RESIGNATION.

MISS M. ROSEVEAR writes from the Infirmary, Newton Abbot, in reference to the statement in our columns that "a nurse who had been at the infirmary for six weeks tendered her resignation, and gave as the reason that she understood that she was engaged as a nurse, and not as a lunatic attendant." The explanation of the workhouse matron was that an imbecile became ill and was removed to the infirmary, and had to be attended by the nurse. We add, by way of comment, "If this is all, the nurse has a poor excuse for leaving." Miss Rosevear, the nurse in question, now sends us a copy of the letter which she addressed to the Newton Abbot Board of Guardians, but which, she states, was not read:—

Newton Abbot Union Infirmary,
August 12th, 1903.

TO THE BOARD OF GUARDIANS.

*Ladies and Gentlemen,*—

I see that the master has explained the case I referred to in my resignation as a simple case from the female imbecile ward. Had that been so there would have been no need for my complaint. I therefore feel that it is only fair to myself that the exact details may be fully known. The case I believe came straight from the padded room, having several bruises about her body and a black eye. She was fairly quiet at first, but gradually became very violent, and as I only had as assistant an old inmate over 70 years of age, who said it was beyond her strength, I went to the sister (who had retired much earlier than usual) and asked for further help, but was refused, and told I should have to put up with the patient, which I did. But I said that I could not hold myself responsible another night, having besides this a male ward to attend to which I was bound to neglect with such a patient. I am not, as I stated before, a lunatic attendant, nor should I like to be, if the scratches, bruises, and blows that I received are the result of just a simple case from the imbecile ward, which I find is now certified and sent to an asylum.

Pardon me for entering into details.

I am, Ladies and Gentlemen, yours obediently,
M. ROSEVEAR.

THE ANGLO-AMERICAN NURSING HOME AT ROME.

"AN ENGLISH NURSE" writes:—A recital of my experience in connection with the Anglo-American Nursing Home at Rome may be of use. From last November till April I nursed the same patient. As it was a nerve case I had very hard duty, doing day and night work together. In consequence, being very run down, I wrote to the matron at the beginning of April that I felt ill, and, having worked uninterruptedly for six months, asked if she would kindly make arrangements, as the season was over at the beginning of May, to let me go home as soon as possible. The matron answered that she could not release me yet, and if my patient did not want me any longer she had other work for me to do. On April 26th I was laid up with a severe attack of typhoid. Throughout my illness I had nothing but a casual letter from the matron about three weeks after I was laid up. The matron neither sent a doctor nor a nurse, nor personally inquired, nor did she even inform my relatives of my illness. We were in a thoroughly Italian mountain place, without reliable doctor and chemist, and if it had not been for my patient happening to be a nurse herself I cannot imagine what the consequences would have been. The matron did ask, after she was called up through the telephone, whether I could be moved; but, as is generally the case with typhoid, it was out of the question, so that my ex-patient had to take the entire charge and responsibility. Towards the end of May it was so hot that the doctor suggested change of air, and wrote a certificate accordingly, adding that I was not fit for work during the two following months. My ex-patient went to Rome to the matron on purpose to inform her that she was leaving for Germany with me on the following day, May 21st. After a most trying journey I naturally had a severe relapse, and was under treatment throughout the whole of June and part of July. A few days after my arrival in Germany I received a letter from the matron blaming me specially on account of leaving Italy, saying that I had broken my contract. The contract one signs for the institute runs, according to the paper, "till the end of the season," no special date or month being given. As it happened, the Roman doctor who treated me assured me that the season was quite over at the end of April, and that the last visitors were only delayed through the German Emperor's visit in the first days of May. As I was ill till towards the end of May, and was not allowed to work for two more months after, I am not aware of my having broken the contract, especially after duly informing the matron of the state of affairs. I have also to mention that the matron and the committee withheld my return fare, which they ought to have paid according to the contract, as well as payment for any expenses incurred through my illness whilst being in their service. I should like very much that the neglect of an English nurse by the Anglo-American Nursing Home should be made known to the English public, especially as it was a case of illness whilst being on duty for an institute.

### Appointments.

CITY FEVER HOSPITAL, BRADFORD.—Miss Evelyn Frost has been appointed head nurse. She was trained at Brownlow Hill Infirmary, Liverpool, and has since been charge nurse at the Fountain Hospital, Tooting, London, and sister of the Army Nursing Service at Netley, Portsmouth, and Winchester Military Hospitals.

KEIGHLEY UNION INFIRMARY.—Miss S. M. Edwards has been appointed charge nurse. She was trained at the Salop Infirmary, Shrewsbury, and afterwards was charge nurse at the Royal Chest Hospital, City Road, North-Western Fever Hospital, and the Chester Isolation Hospital, for various periods.

LAMBETH WORKHOUSE INFIRMARY.—Miss E. M. Bumpus has been appointed sister. She was trained at Paddington Green Children's Hospital and the Royal County Hospital, Ryde, Isle of Wight, where she was afterwards sister.

SOUTHWARK INFIRMARY.—Miss Isabel Kemp has been appointed second-assistant matron. She was trained at the General Infirmary, Northampton, and held subsequently the posts of sister at the Hospital, Sutton, and ward sister and night superintendent at the Southwark Infirmary.

WISBECH UNION WORKHOUSE INFIRMARY.—Miss Louisa Payne has been appointed superintendent nurse. She was trained at St. Luke's Hospital, Halifax Union, and was charge nurse at the Bolton Union Infirmary, and afterwards engaged in private nursing.

WOOLWICH INFIRMARY. — Miss Annie Tighe has been appointed assistant matron. She received her training at St. Olave's Infirmary. She has since been charge nurse at Darenth Fever Hospital, private nursing, district nursing, and head nurse at Hackney Infirmary, having done assistant matron's duties. She holds the L.O.S. certificate.

### Presentations.

HACKNEY INFIRMARY.—Miss A. E. Tighe, before leaving, was presented with a very beautiful inkstand, blotter, note tablet, fountain pen, and also pair of handsome candlesticks, the gifts of the doctors, matron, chaplain, and nursing staff. The presentation was made by the matron, who made a touching reference to the high esteem in which Miss Tighe was held by all who knew her, and the deep regret at her departure from them.

### Echoes from the Outside World.

### The King at Vienna.

On Monday evening the King arrived at Vienna on his State visit to the Emperor Francis Joseph, and met with a most enthusiastic reception. The streets were gaily decorated, Venetian masts having been set up in great numbers over the entire route from the station, and at various points in the Ringstrasse trophies and triumphal arches, draped in the Austrian and British national colours, were erected. The guest chambers at the Hofburg, which his Majesty occupies during his stay in the Austrian capital, are decorated in a sumptuous manner, splendid exotic plants and flowers having been placed in many of the apartments of the palace.

### Funeral of Lord Salisbury.

The remains of Lord Salisbury were laid to rest at Hatfield on Monday afternoon. He was buried as simply as if the obsequies had been those of one of his own cottagers. On the coffin were two magnificent wreaths. One, composed of lilies, roses, orchids, stephanotis, and gardenias, was from the King, and was inscribed, "As a mark of deepest regard, greatest respect, and sincere friendship, from his Majesty the King." The other, in the form of a cross, was from the Queen, and bore the following inscription in her Majesty's own handwriting:—"To the memory of Lord Salisbury, universally loved, and mourned as one of England's best and greatest statesmen.

We think at first that home is heaven,
We learn at last that heaven is home.

Alexandra."

The mourners included the sons and daughters of the deceased statesman and Mr. Arthur Balfour; and the Archbishop of Canterbury, the Bishops of Rochester and Colchester officiated. A memorial service was held in Westminster Abbey simultaneously with the ceremony at Hatfield.

### The Macedonian Rising.

The news from the Balkans continues to be of a serious character. Last week an express train was blown up by dynamite halfway between Adrianople and Constantinople, six persons being killed and fifteen injured. The Macedonian leaders assert that 2,000 men have lately crossed the frontier, and that the insurgents under arms in Macedonia and Adrianople number from 12,000 to 15,000.

### New Colonial Governors.

The successor to Lord Northcote, who vacates the Governorship of Bombay in order to undertake the duties of Governor-General of the Commonwealth of Australia, is Lord Lamington, who in his early days as Mr. Cochrane Baillie, was assistant private secretary to the late Lord Salisbury. Born in 1860, he sat for North St. Pancras until the death of his father in 1890. From 1895 to 1901 he was Governor of Queensland, marrying the year of his appointment the Hon. Mary Houghton Hozier, youngest daughter of the first Lord Newlands. The King has also approved the appointment of Sir Henry Blake, Governor of Hong Kong, to be Governor of Ceylon, in the place of Sir J. West Ridgeway, whose term of office in that colony will shortly expire. Sir Henry Blake, who was born in 1840, has been Governor of Newfoundland, Bahamas, and Jamaica. He was also one of five specially-appointed resident magistrates in Ireland.

### Marriage of Jan Kubelik.

Last week Jan Kubelik, the famous Bohemian violinist, was married at Debrezin, Hungary, to the Countess Marianne Csaky-Szell, who is described as a strikingly handsome young woman and is a near relative of Herr von Szell, the Premier of Hungaria. The romantic story of the betrothal has already been told—how the Countess, then a widow of 21, Kubelik at the time being 19, fell in love with him, and the attraction being mutual, they became engaged, waiting only until Kubelik was of age to be married. Some splendid wedding gifts have been received by the young couple, who are spending the greater part of the honeymoon at Marienbad.

### A Railway to Lyme Regis.

The opening of a new railway between Axminster and Lyme Regis, which took place last week, is an event of considerable interest, and cannot fail to increase the number of visitors to one of the most delightful, as well as one of the most healthy, watering-places on the Dorset coast. With beautiful sands, picturesque scenery, and many other advantages, Lyme only needed the line which now, thanks to the South-western Railway Company, connects it directly with the terminus at Waterloo, to secure it greatly-extended popularity. The new railway line is six and a half miles long, with one intermediate station, and a train leaving London at nine o'clock reaches Lyme Regis at 1.43. There were numerous festivities at the opening, and while the Town Councils of Lyme and Axminster lunched together at Lyme, the children of the town were very considerately allowed to travel over the line free of charge.

### Alpine Disasters.

Last year it was said that the "record" in Alpine disasters had been reached, but this season has been yet more fruitful in tragedies. Amongst recent victims the Rev. S. Hartley, of Exton, who was staying at Pontresina with his wife, on his honeymoon, left with two guides one evening for the Piz Crast Aguzza. All went well till the summit had nearly been reached next morning, when one of the guides felt a violent pull on his rope, which caught on a jagged rock. It parted, and the guide fell a considerable distance, but was able, though badly injured, to get back to the Hut where Mrs. Hartley had gone to meet her husband, and to tell her the sad news that both the other members of the party had been precipitated thousands of feet on to the glacier below. A short time previously a young American Alpinist, with a guide, endeavoured to ascend the Pizzo Norteratsch. When in the middle of the well-known steepest ice-slope, the centre suddenly caved in, forming a crevasse over 150 feet deep. The American gentleman was found alive and almost unharmed, but the guide is badly injured internally. For 25 years this slope has been crossed almost daily and no mishap has ever happened there before.

### Solitude én Réclusion.

The punishment inflicted upon Madame Humbert and her husband is not so light as people in England suppose. It appears that if the conviction is upheld, and the sentence of five years' réclusion, a solitary confinement as it is understood in France, is carried out, the convicts will have a terrible time. The strictest silence is enforced, books are denied, the most complete idleness is enforced, and the half-hour's exercise which is allowed daily is taken subject to a hood being worn which covers everything except the eyes. The prisoner is forbidden even to speak to the warder in charge.

### A Book and its Story.

### NEW NOVEL BY THE AUTHOR OF "THE SILENCE OF DEAN MAITLAND."*

Maxwell Gray's latest novel is written with the fine finish that marks the best writings of a school now considered somewhat out of date. It is one from which up-to-date writers could learn something. The minute care bestowed on characters and scenes, and the leisurely pace which marks the development of the story, are in favourable contrast to the methods of many "modern" novelists.

The hero, Richard Rosny, strong enough in character, is handicapped from the outset by the possession of a mother. Hysterical and emotional, Mrs. Rosny, whose first husband died when Richard was a boy, soon consoled herself for his loss by marrying again. Pretty and fascinating as she was in spite of her unbalanced temperament, a stepfather for Richard was easy enough to secure. The first chapter opens to the sound of her second wedding bells. "The air of a still autumn day trembled to the music of church bells joyously, even recklessly, rung, peal trembling over peal, change clashing upon change in a sweet, mad jangle, a very tumult of exaltation. Joy bells, the villagers called them." "'Why *joy* bells?' Richard asked with a choking sigh. He stood at the gate in the afternoon sunshine, his lips pressed together, his face in a frown, his feet firmly planted, and chin high. . . . Joy bells." Oh, yes; *she* was happy enough. All poor nine-year-old Richard knew was that the happiness he once had was gone. He thought of a few months back, when he had been all-in-all to the worshipped mother, whom in his masculine condescension he regarded as a frail, feminine thing in need of protection. He had been her companion, counsellor, friend. He heard all her grievances, knew when her headaches were coming, draw down the blinds and brought her cups of tea without asking, and stepped about the room without a sound. "Richie is so companionable, I need no other society," he heard her tell people. "Richie is so useful, I could not possibly send him to school. To separate us would break both our hearts."

The memory of his own father, who died when he was four years old, had been constantly renewed by his mother. In the early years of her widowhood she had poured into his half-heeding ears "feelings, regrets, and complaints, from sheer necessity of speech and absence of any other confidant, and, afterwards, from habit and want of consideration. A cheery smile, a strong, kind voice, arms that caught and tossed or carried him was all he could recall of the man whose picture still smiled from the wall, beneath the sword that Richard regarded with silent reverence and secret fascination. He felt, in his dim childish way, that a great wrong had been done the cheery sailor, who smiled on, unsuspecting, in his frame, while others usurped his rights and reigned in his stead. The whole thing was so complicated." Richard entered the Navy. Step-brothers and sisters were added to the home circle. His stepfather, whom he found at first kindly indulgent, if not over-discreet in his attentions, became strangely altered in appearance and manner as time went on. Even as a schoolboy it was apparent to him that something was wrong somewhere, and the conclusion arrived at was that marriage was an eminently unsatisfactory condition of life. "During the holidays the first hint of discord between husband and wife became perceptible. Richard had found his mother crying more than once, and connected the tears with Belton's increasingly frequent absences from home. . . . Little as a boy observes such things, he considered and wondered at the depreciating and contemptuous manner in which Belton habitually alluded to his wife, and was infinitely perplexed both by the bitterness of his mother towards her husband's shortcomings and the indignation with which she resented the faintest aspersion cast upon him, by the passionate fondness and equally passionate disapproval she constantly expressed for him. Nor did he know which he resented most—his stepfather's depreciation of his mother, or her too outspoken devotion to her husband." Too soon Richard finds out that his stepfather is an utterly worthless character, whose moral perceptions, never of the finest, had become hopelessly blunted by the morphia habit.

At seventeen Richard fulfilled the promise of his boyhood. He had grown into a smart young officer and had already distinguished himself at sea. "He had twice saved a life and had been in command under fire." He found when home on leave that his easy, good-natured stepfather was uncertain in temper, gay and morose by turns, that the children would fly from his step, though at times he spoiled them hopelessly. The advent of the sailor brother always brought joy into the troubled household. The children made a willing slave of him, and his mother turned to him for sympathy. He read suffering, repressed anxiety, and terror even in her face, and, as usual, she did not hold back the recital of her miseries, real or self-imposed, from him. At this time she was suffering, he could see, unusually. The domestic discomforts bored and oppressed him, but his mother's daily assertion that he was "her comfort and mainstay" was always a joy to hear. "Richie, darling, I look to you for comfort. And what I have suffered during these cruel years!" Belton had left home on the morning of this particular day, called by business to Paris—a not unusual summons. In his absence Richard and his mother sat later than usual. They had much to hear and to say. This is a glimpse of the two after the children have left them:—"His mother had broken away from the narrowing meshes of personal feeling; Richard listened, happy at her escape from the turbid element of family worries. All was hushed in the pleasant silence of an exquisite autumn night; one shaded lamp near the glowing hearth left the greater part of the room in a darkness broken by the white glimmer of moonlight from tall, uncurtained windows, through which pale stars and moon-steeped stretches of field and woodland were visible. Edith laughed out like a child at something he said; she looked young and attractive, charmingly dressed, with fresh roses in her corsage." Their happy talk was suddenly arrested by the sound of wheels passing under the pine-trees near the house. The door-bell clanged through the house, bolts and bars were shot back, and the door thrown open to disclose a carriage with a jaded horse and a driver walking up and down by its side.

"What's up?" said Richard briskly. The answer was quickly given. The driver, pointing to his fly, intimated that his fare having lost his way he had picked him up and brought him home.

Richard saw at a glance who it was. His mother with ashen face stood watching him, as between them he and the driver carried in the senseless body. "Go upstairs and leave him to me, Muff," he said gently. "Oh, Richard," sobbed the wretched wife, "this is worse than widowhood." "Worse than those joy bells," Richard thought. The story of Richard Rosny must be read—read at leisure to be enjoyed.

* "Richard Rosny." By Maxwell Gray. (Wm. Heinemann. 6s.)

### For Reading to the Sick.

"COME UNTO ME."

I HEARD the voice of Jesus say,
"Come unto Me and rest;
Lay down, thou weary one, lay down
Thy head upon My Breast."
I came to Jesus as I was,
Weary, and worn, and sad:
I found in Him a resting-place,
And He has made me glad.

I heard the voice of Jesus say,
"Behold, I freely give
The living water, thirsty one;
Stoop down, and drink, and live.
I came to Jesus, and I drank
Of that life-giving stream;
My thirst was quench'd, my soul revived,
And now I live in Him.

I heard the voice of Jesus say,
"I am this dark world's Light;
Look unto Me, thy morn shall rise
And all thy day be bright."
I look'd to Jesus, and I found
In Him my Star, my Sun;
And in that Light of life I'll walk
Till travelling days are done.

*McNeale.*

What is it to be saved? It is to have that light of the heart, that strength of the will, that eager purity of the affections, by the force of which we breast the waves of sorrow, sustain ourselves with meekness under the strain of success, and in the darkest hours, as in the brightest moments, do not fail in unselfishness and truth.

What is it to be saved? It is to rise out of the ruts of convention; it is to strangle the treachery of self; it is to have the clear eye and spiritual understanding of the inhabitant of Eternity, to see the beauty of that light that "never shone on sky or sea," the light of perfect human goodness reinforced and purified by heavenly love; to be advancing in fitness to play our part as citizens of that blessed commonwealth which is quickly coming—"the New Heaven and the New Earth wherein dwelleth Righteousness." In one word, it is to have the heart of a man, as his Creator conceived him, pure, tender, and loving; it is with that heart to love God supremely, perfectly; and in God to lose self in love for others—*that* is to be saved!

*Canon Knox Little.*

I could not do without Thee,
O Jesus, Saviour dear;
E'en when my eyes are holden
I know that Thou art near;
How dreary and how lonely
This changeful life would be
Without the sweet communion,
The secret rest with Thee!

I could not do without Thee;
No other friend can read
The spirit's strange deep longings,
Interpreting its need;
No human heart could enter
Each dim recess of mine,
And soothe, and hush, and calm it,
O Blessed Lord, but Thine.

*F. R. Havergal.*

### Notes and Queries.

FOR REGULATIONS SEE PAGE 257.

*Constantinople.*

(236) Will you kindly tell me if there is a hospital at Constantinople, and to whom I should apply for a vacancy there as nurse?—*F. B.*

Apply to the Matron, the British Hospital, Constantinople.

*Maternity.*

(237) Are there any hospitals where, in return for services, nurses are trained in maternity nursing either during or after other training? I have had five years' hospital work.—*M. M. D.*

The Clapham Maternity Hospital, Jeffreys Road, Clapham, S.W., offers nurses, already medically and surgically trained, two months' training for a reduced fee. Apply the Hon. Secretary. Several training schools under the Poor Law include maternity in their course of training. For these see "The Nursing Profession: How and Where to Train."

*Massage.*

(238) I have taken a certificate in Massage and Electricity from the National Hospital for the Paralysed and Epileptic. Can you tell me how to set about getting work? 1. Should I call upon doctors, or advertise? 2. If I advertise, which papers besides THE HOSPITAL are the best? 3. Should I wear uniform? 4. Is it any advantage to belong to the Society of Trained Masseuses?—*L. S.*

1. Both. 2. The medical and local journals. 3. It is entirely a matter of personal taste. It is not necessary. 4. Certainly.

*Hospital Training.*

(239) Are there any Children's Hospitals where no fee for training is required? What is the youngest age at which probationers are taken?—*M. B.*

You will find full particulars in "The Nursing Profession: How and Where to Train." Twenty, or twenty-one, is the usual age for probationers to enter Children's Hospitals.

*Gynæcological Examinations.*

(240) I should be much obliged if you can tell me when and where the Gynæcological Society's Examinations are to be held; and what text books cover the course of instruction.—*G. F.*

Send a stamped and addressed envelope to Dr. Aarons, 14 Stratford Place, London, W., for all particulars.

*Stewardess.*

(241) *Nurse E. E.* will be obliged if the Editor will kindly inform her (1) if trained nurses are employed by the P. and O. Company, or by any large company running passenger boats. 2. To whom should she apply for information respecting the duties of stewardess on board the Cunard or P. and O. Line, and the probability of vacancies for the same?

1. Trained nurses are not at present employed by the large shipping companies. But there is a movement on foot with that object in view. 2. Meanwhile, you had better apply to the secretaries of the respective companies, or to Miss Penn, The Cottage, New Shoreham, Sussex.

*Padding Truss.*

(242) I would be obliged for information as regards padding a truss. My husband wears one, and I have tried chamois leather, cotton wool, etc., but it lasts only a week, and he complains of irritation.—*H. G.*

Chamois leather, powdered each day with French chalk, is usually the best material for padding a truss. Judging from the amount of irritation, it may be that the truss does not fit quite well; if so a medical man should be consulted.

*Enema.*

(243) Will you kindly tell me the correct pronunciation of the word "Enema"?—*G. H. B.*

As neither etymology nor general usage can decide the correct pronunciation, the word may be pronounced ennema or eneema as you please.

**Standard Nursing Manuals.**

"The Nursing Profession: How and Where to Train." 2s. net; 2s. 4d. post free.

"Nursing: Its Theory and Practice." (Revised Edition). 3s. 6d. post free.

"Surgical Ward Work and Nursing." (Revised Edition). 3s. 6d. net.; 3s. 10d. post free.

"Practical Handbook of Midwifery." (New Edition). 6s. net; 6s. 3d. post free.

"Notes on Pharmacy and Dispensing for Nurses." 1s. post free.

"Fevers and Infectious Diseases." 1s. post free.

"The Art of Massage." (New Edition). 6s. post free.

## Figures and Tables

**Fig. 1. f1:**
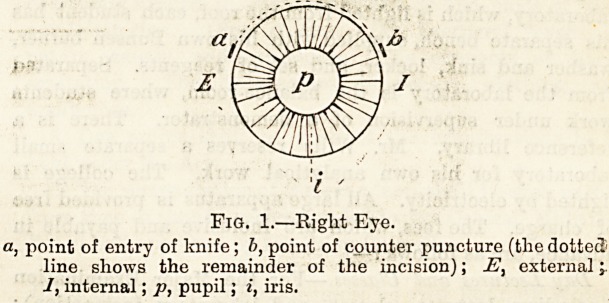


**Fig. 2 f2:**